# The Anti-Cancer Potency and Mechanism of a Novel Tumor-Activated Fused Toxin, DLM

**DOI:** 10.3390/toxins7020423

**Published:** 2015-02-04

**Authors:** Dejun Sun, Miaonan Sun, Wenhe Zhu, Zhiding Wang, Yuefei Li, Jie Ma

**Affiliations:** 1 Department of Biomedicine, Institute for Regeneration Medicine, Jilin University, Changchun 130021, China; E-Mails: sunmn1987@hotmail.com (M.S.); wzdfjfn@126.com (Z.W.); liyfjlu1989@126.com (Y.L.); 2 Department of Biochemistry, School of Basic Medicine, Jilin Medical College, Jilin 130000, China; E-Mail: zhuwenhejl@163.com; 3 School of Pharmaceutical Sciences, Jilin University, Changchun 130021, China

**Keywords:** tumor-activated, fused toxin, disintegrin, melittin, anti-cancer

## Abstract

Melittin, which acts as a membrane-disrupting lytic peptide, is not only cytotoxic to tumors, but also vital to normal cells. Melittin had low toxicity when coupled with target peptides. Despite significant research development with the fused toxin, a new fused toxin is needed which has a cleavable linker such that the fused toxin can release melittin after protease cleavage on the tumor cell surface. We describe a novel fused toxin, composed of disintegrin, uPA (urokinase-type plasminogen activator)-cleavable linker, and melittin. Disintegrin is a single strand peptide (73 aa) isolated from *Gloydius Ussuriensis* venom. The RGD (Arg-Gly-Asp) site of disintegrin dominates its interaction with integrins on the surface of the tumor cells. uPA is over-expressed and plays an important role in tumor cell invasiveness and metastatic progression. The DLM (disintegrin-linker-melittin) linker is uPA-cleavable, enabling DLM to release melittin. We compared binding activity of our synthesized disintegrin with native disintegrin and report that DLM had less binding activity than the native form. uPA-cleavage was evaluated *in vitro* and the uPA-cleavable linker released melittin. Treating tumors expressing uPA with DLM enhanced tumor cell killing as well as reduced toxicity to erythrocytes and other non-cancerous normal cells. The mechanism behind DLM tumor cell killing was tested using a DNA ladder assay, fluorescent microscopy, flow cytometry, and transmission electron microscopy. Data revealed tumor cell necrosis as the mechanism of cell death, and the fused DLM toxin with an uPA-cleavable linker enhanced tumor selectivity and killing ability.

## 1. Introduction

Traditional chemotherapy drugs and radiotherapy is based on the rapid proliferation of cancer cells [1,2], and although these treatments increase patient survival and destroy tumors, these methods also harm other dividing cells, such as epithelial and hematopoietic stem cells [3,4]. Thus, tumor-targeted toxins may be helpful for developing novel anti-cancer therapeutics. Targeted toxins contain a cell-targeting domain and a cytotoxic agent, connected directly with or by way of a suitable linker [5,6,7,8], which must eventually be cleaved to release the cytotoxic agent [9,10,11,12]. uPA is a tumor-specific protease highly expressed in several malignant tumor cells but rarely present in normal cells. uPA can be concentrated and activated by uPAR, which resides on tumor cell surfaces, and this can degrade the uPA-cleavable complex. Finally, the uPA cleavage site can be used as a peptide linker to increase affinity and specificity of targeted toxins.

Integrins are cell surface receptors needed for cell adhesion and migration, and they are comprised of heterodimeric α- and β-subunits [13,14] that control cell-signaling pathways, cell polarity, and force generation. Integrins are also involved in matrix-degradation, which is required for cell migration and invasion. Thus, integrins are potential targets for developing anti-cancer therapies [15,16,17,18]. Disintegrins, found in snake venom, can target to integrins with strong affinity and block the RGD motif of the integrin functional domain. Disintegrins can inhibit several aspects of tumor cell behavior *in vitro* and *in vivo*, such as adhesion, migration, invasion, metastasis, and angiogenesis, by binding to integrins αvβ3, αvβ5, and/or α5β1 [19,20,21]. Contortrostatin, a homodimeric disintegrin (64 residues in each chain) from southern copperhead snake venom, has impressive inhibitory effects in models of breast, ovarian and prostate cancers, and it is a representative disintegrin with an RGD motif [22,23,24]. An anti-cancer nanoparticle containing contortrostatin has been constructed and it can inhibit tumor growth in two breast carcinoma cell lines, compared with nanoparticle controls or contortrostatin control alone [25]. Ussurin, a disintegrin from *Gloydius Ussurinsi’s* venom, is a small disulfide-rich peptide (6800 Daltons) with properties similar to contortrostatin (has 65 amino acids and an RGD motif).

Melittin (26 amino acids) is a major peptide component of bee venom with high cancer-fighting potency [26]. Melittin is cytotoxic to cancer cells, inhibiting cell growth and inducing caspase activation, leading to apoptosis and necrosis [27,28,29,30,31,32,33]. Melittin is not only toxic to diverse tumors, but is also vital to normal cells. It can cause hemolysis by disrupting erythrocyte membranes [34,35] via inserting into lipid bilayers to form tetramer aggregates as ion outflow channels [36]. Melittin has low lytic activity when coupled with target peptides such as an immunoconjugate of melittin, an adenovirus-melittin, a melittin-avidin conjugate, and a RGD-melittin conjugate [30,37,38].

Previously, we constructed a tumor-activated conjugate DLM and expressed it in *Pichia pastoris*. After optimization of growth and induction conditions, the DLM yield was 160 mg/L (95% purity). DLM had less hemolytic bioactivity, allowing for intravenous drug delivery. DLM had low cytotoxicity to uPA non-expressing HEK-293 cells and strong cytotoxicity against uPA-expressing cells A549 [39].

We compared the biological activity between DLM and native disintegrin to evaluate uPA-cleavage, and we assayed tumor cytotoxicity as well as safety for erythrocyte and human normal cells. Finally, we investigated how DLM kills tumor cells using a DNA ladder assay, fluorescent microscopy, flow cytometry and transmission electron microscopy (TEM).

## 2. Results

### 2.1. Disintegrin Is Released from DLM by uPA Cleavage

**Figure 1:**
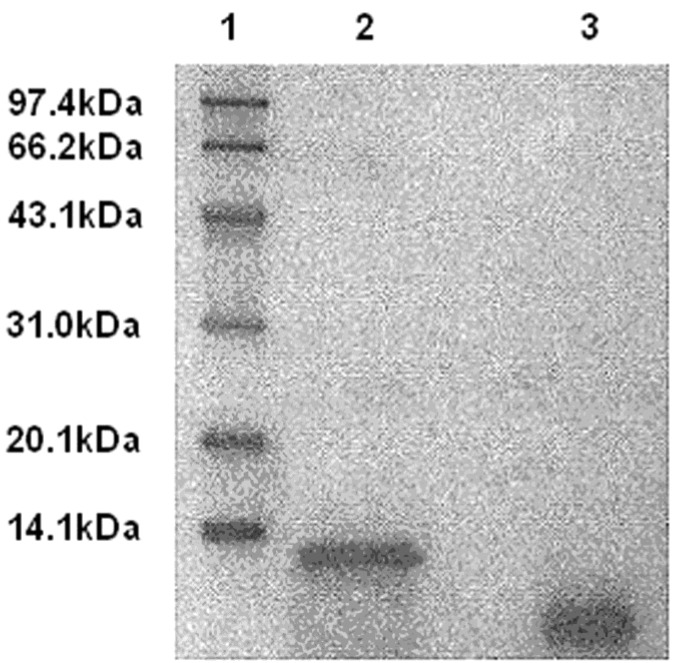
Degradation of DLM by uPA. Lane 1: Protein standard; Lane 2: DLM (12,000 Da, Table S1); Lane 3: DLM cleavage by uPA (band ~10,000 Da, and 73 + 16 aa), indicating that DLM can be cleaved by uPA.

The DLM can be cleaved by uPA specifically to release the disintegrin domain (Figure 1).

### 2.2. Hemolysis Bioactivity of the DLM

**Figure 2:**
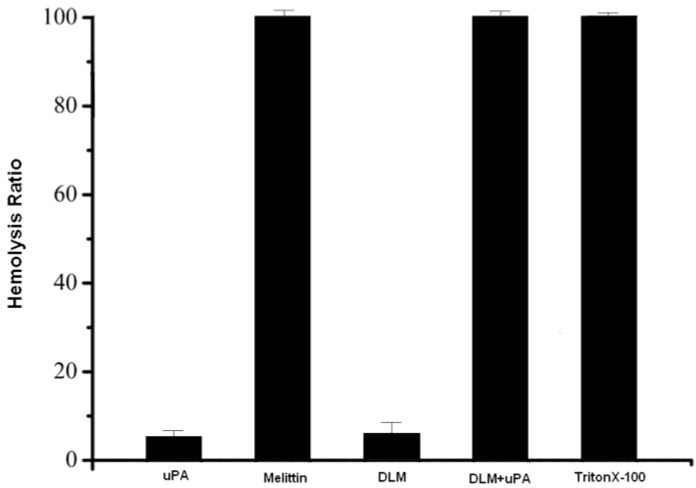
DLM and erythrocytes. Free melittin and DLM activated by uPA are hemolytic, but DLM is weakly hemolytic. Results are expressed as means ± standard error.

In order to show that DLM is less cytotoxic than melittin and that the cleaved DLM is toxic to tumor cells, an erythrocyte hemolysis assay was used. After incubating DLM, DLM + uPA and free melittin at 37 °C for 1 h, DLM was observed to cause minimal hemolysis at 10 μmol/L. When erythrocytes were treated with the same concentrations of DLM + uPA and melittin, significant hemolysis occurred (Figure 2). Free melittin is a nonspecific cytolytic peptide that can rapidly lyses erythrocyte lipid membranes, causing toxicity when injected intravenously. Melittin coupled to DLM, on contrast, may be safely delivered intravenously and melittin is released after selectively targeting tumor cells with little off-target toxicity.

### 2.3. Native Disintegrin and DLM Activity

**Figure 3:**
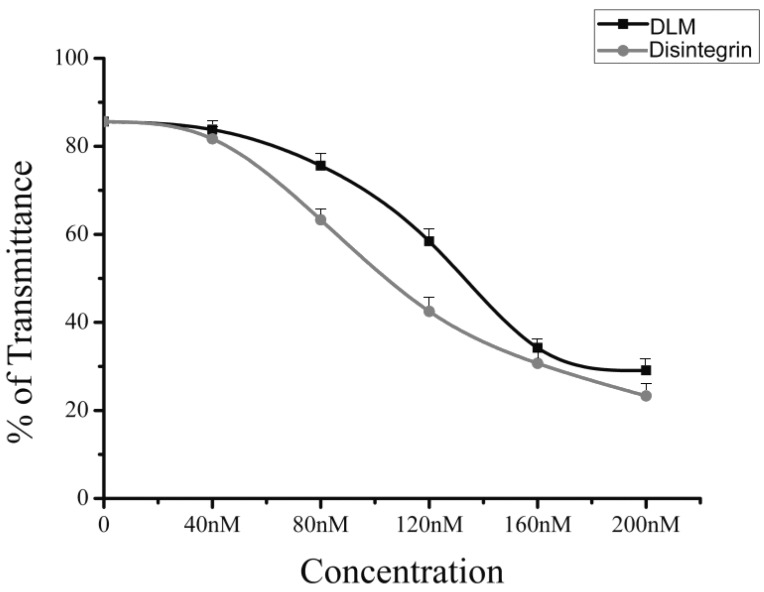
Retention of biological activity of disintegrin in DLM. Results are expressed as mean ± standard error.

The disintegrin domain of DLM retains platelet aggregation inhibitory activity, although this domain is attached to DLM. Intact DLM can greatly inhibit platelet aggregation (IC_50_ of disintegrin attached to DLM = 130 nM, compared to native disintegrin IC_50_ 102 nM; Figure 3). A platelet aggregometer was used to ADP-induced aggregation inhibition. Disintegrin in the DLM complex was the same as PBS with regard to inhibiting platelets. Disintegrin coupled to DLM was stable during prolonged incubation in plasma and had little biological activity.

### 2.4. Binding of Disintegrin to Inactive Platelets

**Figure 4:**
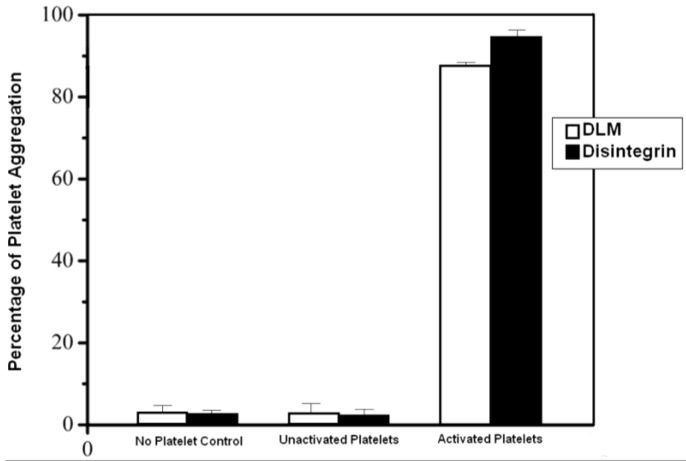
DLM binding to washed platelets compared with disintegrin. Results are expressed as means ± standard error.

Disintegrin and DLM were labeled with FITC and then purified by dialysis and a Sephadex G25 gel column (F/P ratio 0.72). DLM binding to washed platelets was compared with disintegrin under identical conditions (Figure 4). Neither compound adhered to inactivated washed platelets but after ADP activation, both compounds bound to platelets during the 10 min incubation. Thus, disintegrin coupled to DLM can potently bind platelets.

### 2.5. MTT Assay

**Figure 5:**
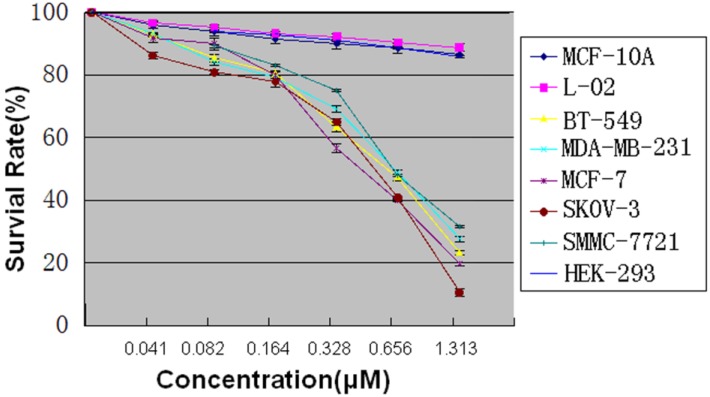
MCF-7 cell survival after treatment with serial dilutions of DLM.

Every tumor cell strain was cultured and treated with DLM and incubated for 72 h (Table S2, Figure 5 depicts OD_490_). Serial dilutions of DLM show a dose-dependent cytotoxicity against BT-549, MDA-MB-231, SMMC-7721, MCF-7, and SKOV-3 cells. DLM killing of HEK293 was low (2.626 μM DLM) and caused cytoplasmic condensation only. Under an inverted microscope, control MCF-7 cells were normal; with 0.164 µM DLM there was significant change. With 0.328 µM DLM cells were swelling, plasma membranes ruptured, and content released into the extracellular area, suggesting necrosis. MCF-7 cells treated with 1.313 µM DLM did not have complete cell structures.

### 2.6. DNA Ladder

**Figure 6:**
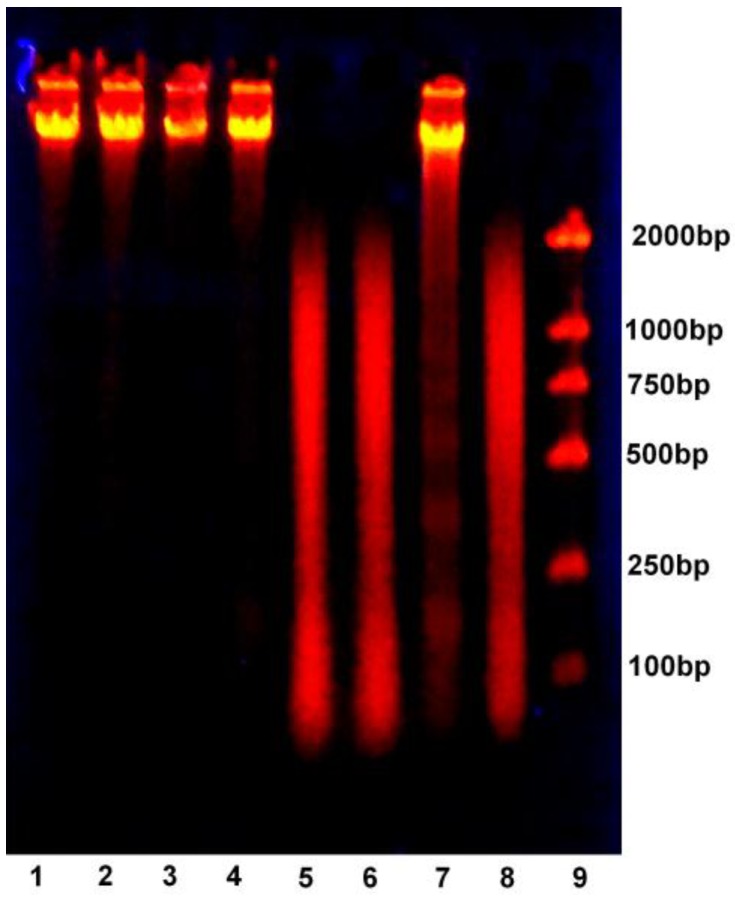
MCF-7 cells after gel electrophoresis fluorescence of DNA after 48 h. Lane 1: Negative control; Lane 2: MCF-7 cells treated with 0.164 μM DLM; Lane 3: 0.328 μM DLM; Lane 4: 0.626 μM DLM; Lane 5: 1.313 μM DLM; Lane 6: 2.626 μM DLM; Lane 7: 10 μM *cis*-platinum; Lane 8: 2.626 μM melittin; Lane 9: DNA marker.

DNA of MCF-7 cells treated with serial dilutions of DLM were observed after gel electrophoresis fluorescence after 48 h incubation. Negative control cells and DLM-treated cells (0.164, 0.328, and 0.656 μM) have a clear band and no laddering. DLM-treated cells (1.313 and 2.626 μM) and melittin-treated cells appear as smears. The *cis*-platinum group has a ladder pattern (Figure 6).

### 2.7. Flow Cytometry and Fluorescence Microscope

**Figure 7:**
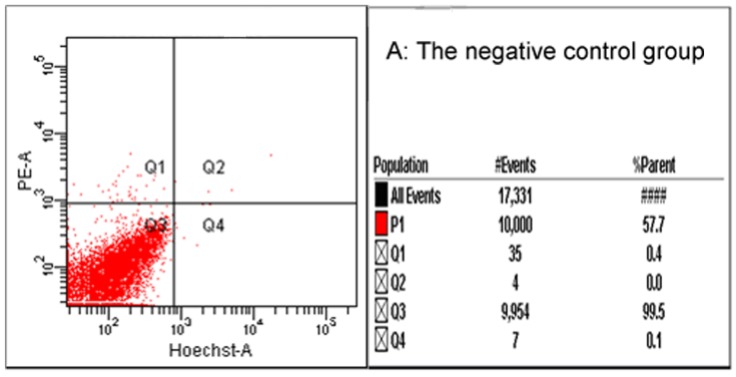

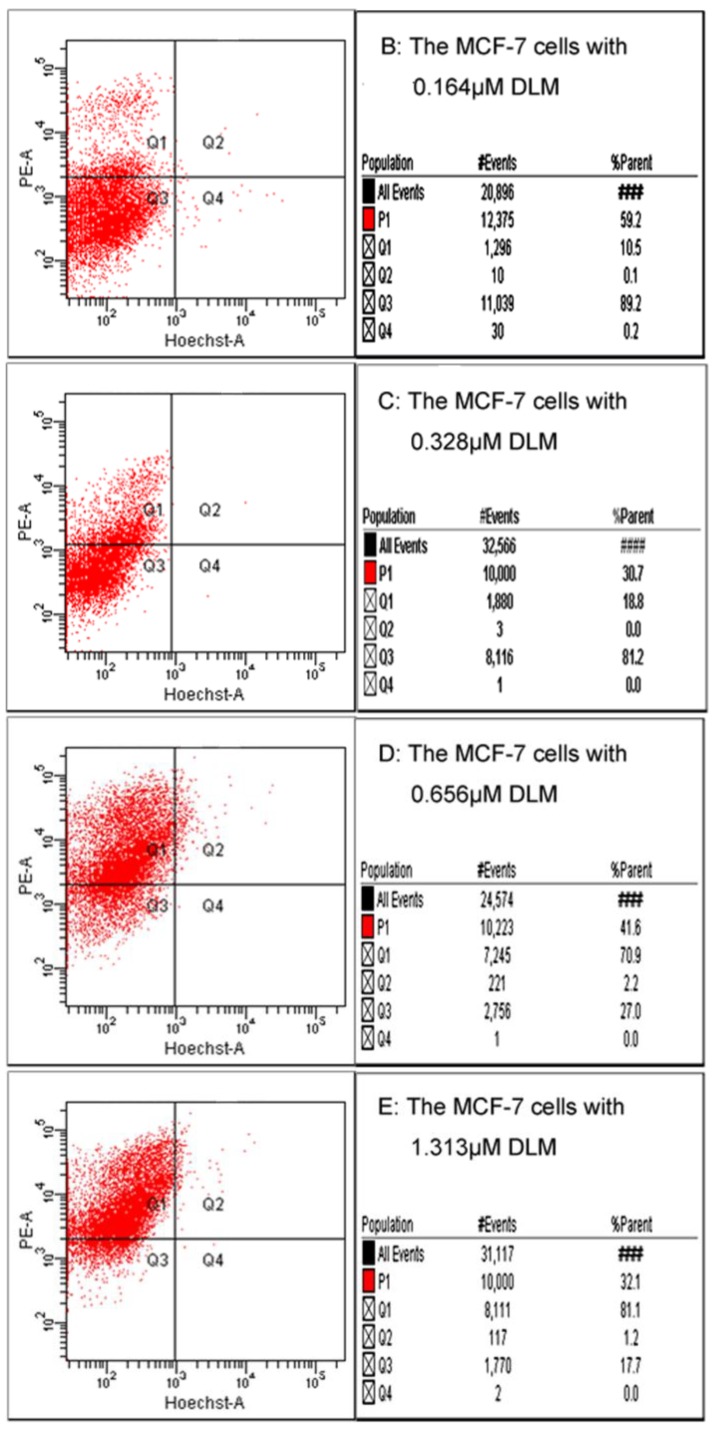
Cytotoxicity of DLM to MCF-7 measured by flow cytometry. (**A**) controls; (**B**) 0.164 μM DLM; (**C**) 0.328 μM DLM; (**D**) 0.656 μM DLM; (**E**) 1.313 μM DLM.

**Figure 8:**
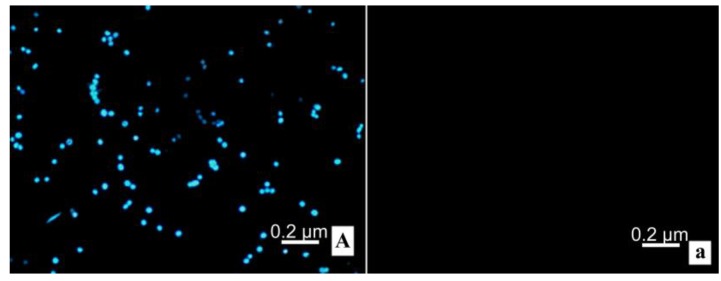

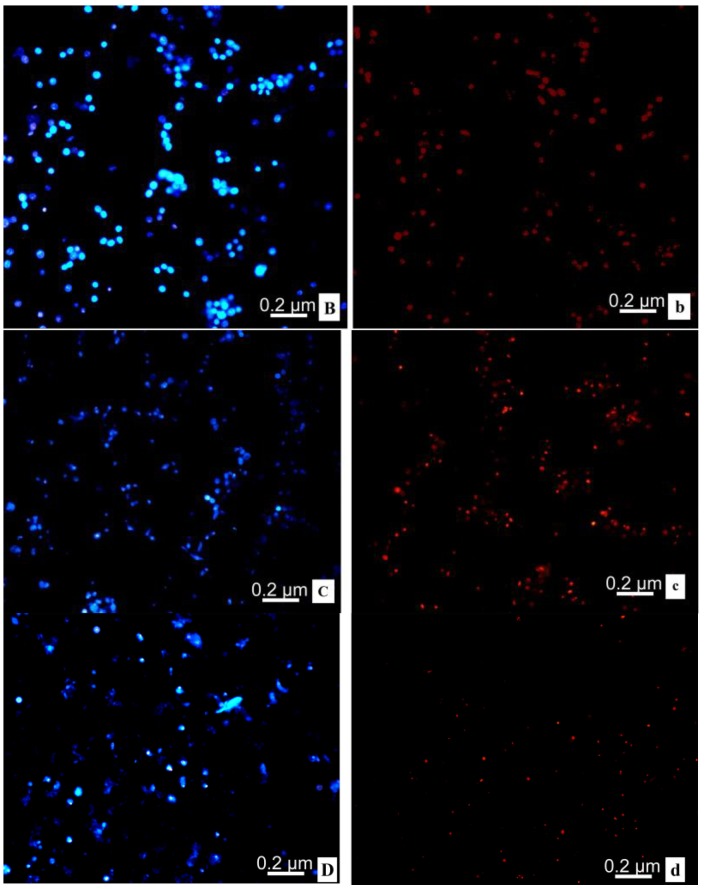
Cytotoxicity to MCF-7 observed under fluorescent microscopy (200×). (**A**,**a**) controls; (**B**,**b**) 0.328 μM DLM; (**C**,**c**) 0.656 μM DLM; (**D**,**d**) 1.313 μM DLM.

Cells are measured with flow cytometry and observed under a fluorescent microscope. Spots mainly appeared in the Q1 section, implying necrosis, which increased with increasing DLM (Figure 7). Under the fluorescent microscope, MCF-7 cells (control) contain blue fluorescence and MCF-7 cells treated with DLM treated have red fluorescence, which increases with increasing DLM dose (Figure 8).

### 2.8. Transmission Electron Microscope; TEM

**Figure 9:**
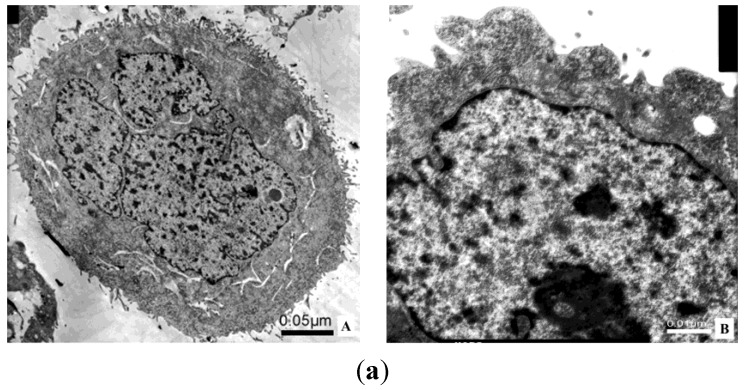

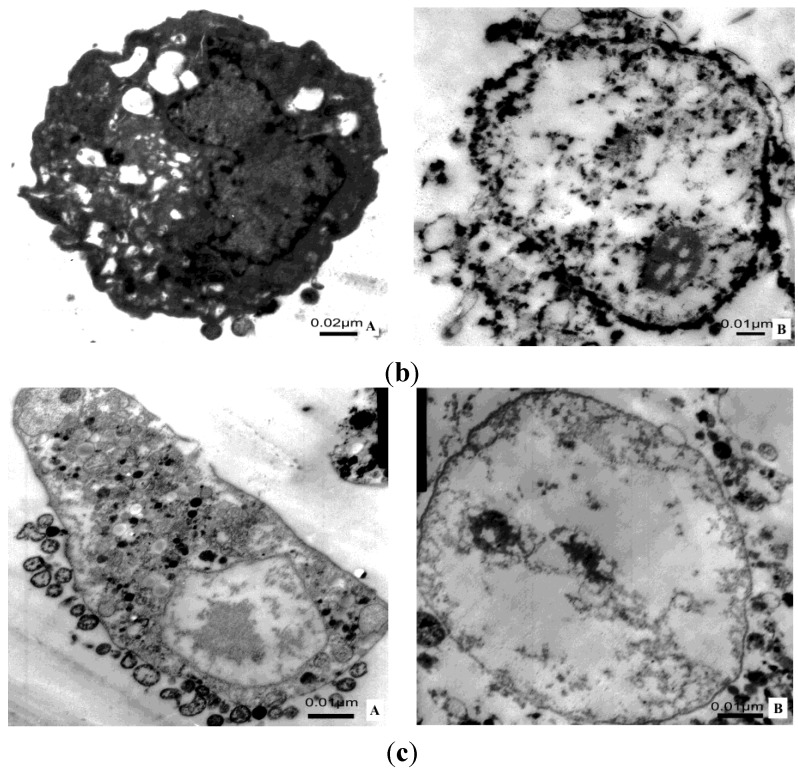
MCF-7 cells under TEM. (**a**) Control MCF-7 cell under TEM. **A**: Cells are larger with microvillus and the nucleus is large and irregular (2000×); **B**: Some cells have free ribosomes (12,000×); (**b**) MCF-7 cells treated with 0.328 μM DLM under TEM. **A**: Cells are smaller and leaky, the cytoplasm has many vacuoles and few lipid droplets (5000×); **B**: The ruptured membrane (indicated by arrow), released contents and degraded organelles are present in the cytoplasm. ER, nuclear matrix, and cytoplasm have vacuoles (7500×); (**c**) MCF-7 cell treated with 0.656 μM DLM under TEM. **A**: Cytoplasm shows membrane rupture (indicated by arrow at top) and the nucleus has vacuoles (indicated by arrow at right). Intact organelles are scarce. (6000×); **B**: Cells are swollen and the nuclear matrix (indicated by arrow) and the cytoplasm have vacuoles (6000×).

MCF-7 cells treated with DLM were necrotic (cytoplasmic membrane rupture, content release, and organelle breakdown). The ER (endoplasmic reticulum) and the nuclear matrix and cytoplasm had vacuoles (Figure 9).

## 3. Discussion

Invasion and metastasis are key to tumor malignancy and these events are complex interactions of host and cells and the extracellular matrix. In this process, the combination of normal epithelial cells and the matrix is achieved by integrins, which are present on epithelial cell surfaces. The ligand is in the matrix. Thus, the integrin family of cell adhesion receptors is vital to initiation, progression and metastasis of malignant tumors and attractive targets for anti-cancer therapy. Due to the affinity of integrin for what, we selected the disintegrin, ussurin, as the carrier because it can deliver the toxin domain, melittin, to cancer cells with high efficiency and accuracy.

Melittin is attached in DLM via a linker to confine its hemolytic activity and to increase safety of delivery. Melittin is released when DLM reaches the target cell and is cleaved by uPA, which is mainly expressed on the cancer cell surface. Then, the cancer cell membrane is ruptured by melittin, and cell death ensues. SDS-PAGE revealed that after DLM treatment with uPA, DLM can be cleaved to release the melittin and disintegrin domains. Intact DLM can inhibit platelet aggregation (IC_50_ 130 nM) and its IC_50_ is higher than native natural disintegrin (IC_50_ 102 nM). Incubation of native disintegrin and DLM, with washed, inactivated human platelets, revealed no inhibition by either molecule but after ADP activation both molecules efficiently inhibited platelets.

We observed that inhibition activity was varied with each cancer cell line and that inhibition was dose-dependent. Under an inverted microscope, dead cells increased as DLM increased. During apoptosis, cells produce DNA fragments of particular lengths and these fragments create a ladder pattern with gel electrophoresis. In contrast, DNA fragments that form necrosis appear as smears with electrophoresis. Control and DLM-treated cells presented as a clear band (no ladder) and DLM-treated cells were smears, indicating that necrosis occurred.

Flow cytometry confirmed that DLM was cytotoxic to MCF-7 cells. Under TEM, normal MCF-7 cells are clear with microvilli, a large irregular nucleus and normal cytoplasmic organelles. After treatment with DLM, MCF-7 cell membranes ruptured, the ER had vacuoles, and organelles were degraded, all indicators of necrosis. DLM may be useful for anti-cancer therapy because it can inhibit cancer cell growth via necrosis and reduce melittin-induced hemolysis and other toxic effects on normal cells. Our work provides a foundation for further exploration of DLM for anti-tumor biological functions and applications.

## 4. Materials and Methods

### 4.1. Cell Lines and Cell Culture

The cells used in our studies were as follows:

(1) SMMC-7721, a human hepatoma cell line,
(2) MCF-7, a human breast carcinoma cell line,
(3) BT-549, a human breast carcinoma cell line,
(4) MDA-MB-231, a human breast carcinoma cell line,
(5) SKOV-3, a human ovarian neoplasm cell line,
(6) MCF-10, a human normal breast cell,
(7) L-02, a human normal liver cell, and
(8) HEK293, a human normal embryonic kidney cell.

All cell lines were maintained in RPMI 1640 medium (Invitrogen Inc., Carlsbad, CA, USA) with 20% fetal bovine serum and 10% DMSO at −80 °C. Cells were cultured in RPMI 1640 medium with 10% fetal bovine serum, 100 units/mL penicillin, and 100 μg/mL streptomycin at 37 °C, in 5% CO_2_.

### 4.2. DLM

DLM was expressed and purified as previously described [39]. The selected recombinant *P. Pastoris* strain pPIC9K/DLM was cultured in BMGY, and induced with 0.5% methanol in BMMY media. After 96 h of culture, the medium was harvested by centrifugation and the supernatant was concentrated by ultrafiltration.

The concentrated solution was loaded onto a SP-Sepharose Fast Flow column, the eluted protein, was loaded onto a Sephadex G 75 column. Purified DLM was a single band on SDS-PAGE and Western blot (95% purity).

### 4.3. Hemolysis Assay of DLM

As described previously [20], DLM was activated by uPA, and uPA and free melittin were incubated with sheep erythrocytes (final concentration 10 μmol/L; final erythrocyte concentration 1% (*v*/*v*)). The suspension was incubated for 60 min at 37 °C with gentle mixing. Samples were then centrifuged and supernatant absorbance was measured at 545 nm. Controls for zero hemolysis (blank) and 100% hemolysis consisted of erythrocytes suspended in PBS and 1% Triton X-100, respectively. Data (means ± SD) were from three independent experiments.

### 4.4. Disintegrin Released from DLM by uPA in Vitro

The fused toxin DLM contained a uPA-cleavable sequence to link carrier and toxin domains. To verify linker cleavage with uPA *in vitro*, a reaction mixture containing 5 µg DLM, 0.5 µg uPA, and 50 µL enzyme buffer (150 mM NaCl, 10 mM Tris-HCl pH 7.5) was prepared. The reactant was analyzed by SDS-PAGE after incubating at 37 °C in a water bath for 60 min.

### 4.5. Assay DLM Activity

The DLM contained equal moles of disintegrin and melittin and the biological activity of disintegrin within the DLM is crucial for a tumor-activated fused toxin. Activity was assessed by measuring disintegrin-mediated inhibition of ADP-induced platelet aggregation.

Briefly, the inhibitory activity of native disintegrin and DLM toward ADP-induced platelet aggregation was measured with a platelet aggregometer (ACL7000, Beckman Coulter Inc., Fullerton, CA, USA) with human platelet rich plasma (PRP). Whole human blood (50 mL), obtained from volunteers and drawn into 5 mL tubes, with 0.1 mol/L sodium citrate. Donors had not received any aspirin-based medications for at least two weeks prior to donation. Sample blood was centrifuged (1000 *g* for 10 min) at 25 °C. The supernatant, PRP, was carefully transferred to a new tube. PRP (1 mL) was placed in a microcentrifuge tube and centrifuged at (8000 *g* for 5 min) 25 °C. The supernatant was removed and retained as platelet-poor plasma (PPP). PPP was then used as a blank to standardize the platelet aggregometer. Inhibition of ADP-induced platelet aggregation was monitored at 37 °C by adding samples (disintegrin and DLM) over a range of disintegrin concentrations (0–200 nM) to PRP 1 min prior to the addition of ADP. Data (means ± SD) were from three independent experiments.

### 4.6. Binding of Disintegrin and DLM to Inactive Platelets

Disintegrin and DLM binding to washed human platelets was evaluated. PRP and PPP were prepared as described above and supernatant (PPP) was decanted, and the inside wall of the centrifuge tube was wiped. Modified Tyrode’s buffer (0.1% glucose, 0.8% NaCl, 0.1% NaHCO_3_, 0.02% KCl, 1% BSA in 10 mM HEPES (pH 7.2)) was added (1 mL/mL of original PRP) and PGE1 was added to a concentration of 2.5 mM. A small amount (1–2 mL) of PPP was set aside for a zero-platelet control in the binding experiments. After addition of Tyrode’s buffer, platelets were resuspended by gently stirring the buffer with a disposable plastic pipette; platelets were gently drawn into and expelled from the pipette until minuscule particles of the original pellet were no longer visible. Pelleting and pellet resuspension were repeated three times, and a final resuspension was in 6 mL modified Tyrode’s buffer without PGE1. Platelets were counted, and washed platelets were used in binding experiment with different forms of disintegrin.

FITC (fluorescein isothiocyanate), native disintegrin and DLM were dissolved in buffer (7.56 g NaHCO_3_, 1.06 g Na_2_CO_3_, and 7.36 g NaCl, in 1 L water), respectively (20 mg native disintegrin or DLM at 2 mL, 7.8 mg FITC at 3 mL). FITC solution was added to native disintegrin or DLM solution dropwise while gently and continuously shaking the protein solution. After FITC solution was added at the molar ratio of 1:2 (DLM or native disintegrin:FITC), the reaction was incubated in the dark for 16 h at 4 °C. The conjugate of the FITC-protein was put into a dialysis bag, and this was dialyzed overnight to separate unbound FITC from the conjugate. Unreacted FITC was removed from FITC-disintegrin or FITC-DLM solution by gel filtration using a gel matrix of Sephadex G25 column (40 × 1.0 cm). After elution with PBS, two peaks formed. The first peak was FITC-protein, which can easily be visualized. The ratio of fluorescein to protein in the conjugates was measured using absorbance at 495 nm (F) and 280 nm (P).

Tubes were divided into two groups, each group had 3 tubes with 200 μL washed platelets (3 × 10^5^ platelets/mL in Tyrode’s buffer) and 1 tube with Tyrode’s buffer as the control, FITC-disintegrin, FITC-DLM were added to the 2 group tubes (final concentration 30 nmol/L). ADP was added to one group as a platelet activator (final concentration 20 μmol/L) and the same volume of Tyrode’s buffer was added to the other group (inactivated platelets). The mixtures were incubated for 30 min at 37 °C. Platelets were pelleted by centrifugation and fluorescence measured by counting fluorescence in both the supernatant and the pellet with a fluorophotometer (λ_ex_ = 500 nm, λ_em_ = 530 nm, slit = 10 nm). Data (means ± SD) were from three independent experiments.

### 4.7. MTT Assay

Cells (SMMC-7721, MCF-7, BT-549, MDA-MB-231, SKOV-3, MCF-10A, L-02, and HEK293) were seeded on 96-well Xat-bottomed plates (3000 cells/well), and incubated at 37 °C under 5% CO_2_ for 24 h. Then cells were treated with serial dilutions of DLM after removing the medium (0.041, 0.082, 0.164, 0.328, 0.656, 1.313, and 2.626 μM). There were six individual wells for each dose. The blank control was the dilution medium without the sample. Then cells were incubated in 96-well Xat-bottomed plates at 37 °C under 5% CO_2_ for 72 h. After incubation, supernatant was removed and cells were washed with PBS (pH 7.4). Cells were incubated with medium containing 20 μL of the MTT solution (5 mg/mL) at 37 °C for 4 h. To dissolve formazan crystals, 100 μL of DMSO was added after removing the supernatant. OD was measured with an ELISA microplate reader at 490 nm. Cytotoxicity was measured with IC_50_. Data (means ± SD) were from three independent experiments.

### 4.8. Flow Cytometry and Fluorescent Microscopy

After 48 h of treatment with serially diluted DLM (0, 0.164, 0.328, 0.656, 1.313, and 2.626 μM) MCF-7 cells were collected (1 × 10^6^ cells). Every tube was washed twice with cold PBS. Medium (1 mL) was added to each tube containing 10 μL Hoechst 33342 (5 µg/mL). This was mixed thoroughly and incubated at 37 °C for 10 min. Cells were centrifuged at 1500 *g* for 5 min at 4 °C and supernatant was discarded. Cells were resuspended in 1 mL buffer (1 μg/mL PMA in RPMI 1640 medium). Then 5 μL of PI was added to each cell suspension and mixed thoroughly. Cells were incubated at room temperature for 10 min. After incubation, stained cells were analyzed with flow cytometry immediately (λ_ex_ = 352 nm, λ_em_ = 400–500 nm for Hoechst; λ_ex_ = 488 nm, λ_em_ => 630 nm for PI).

### 4.9. DNA Ladder Assay

MCF-7 cells were collected (1 × 10^7^) after a 48 h culture with medium and serially diluted DLM as previously described. A necrosis-positive control group received 2.626 μM melittin and an apoptosis-positive group was treated with 10 μM *cis*-platinum. Cells were washed three times with PBS, and then resuspended in 350 mL of lysis solution (50 mmol Tris-HCl, 0.1 mol EDTA, 0.5% SDS) and 3.5 μL (50 μg/mL) of proteinase K. cells were incubated in a water bath (50 °C) for 2 h. Next, equal volumes of phenol-chloroform-isoamyl alcohol (25:24:1) were added to cells and mixed. The mixture was centrifuged at 12,000 *g* for 10 min at 4 °C. Supernatant was discarded and 1/10 volumes of 5 mol/L NaCl and 2.5 volumes of ethanol was added to the cells which were centrifuged at 12,000 *g* for 10 min at 4 °C. Precipitates were washed in 75% ethanol and left to dry at room temperature. Precipitates were re-suspended in 100 μL TE buffer (20 mM Tris-HCl, 1 mM EDTA, pH 8.0) and incubated with 2 μL (10 mg/mL) RNase for 1 h at 65 °C and then separated by 1% gel electrophoresis.

### 4.10. TEM

MCF-7 cells were collected (1 × 10^5^) after a 48 h culture with medium and serially diluted DLM as described previously. Cells were washed with precooling PBS twice and then fixed in 2.5% glutaraldehyde at 4 °C for 2 h. Cells were post-fixed in 1% osmium tetroxide at 4 °C for 1 h and then washed with PBS for 20 min twice. Cells were stained with 0.5% aqueous uranyl acetate at room temperature overnight. To dehydrate slides, they were immersed in 30%, 50%, 70%, and 90% ethanol for 10 min at each percent and then 100% ethanol for 10 min twice. Samples were embedded in Epon812 and cut into ultrathin sections. Sections were collected onto grids and stained with 1% (*w*/*v*) uranyl acetate and lead citrate. Samples were observed under a JEM-1200EX electron microscope (Nihon Kohden Corporation, Tokyo, Japan).

## Supplementary Materials

Supplementary materials can be accessed at: http://www.mdpi.com/2072-6651/7/2/0423/s1.

## Abbreviations

DLM	disintegrin-linker-melittin	TEM	transmission electron microscope
RGD	Arg-Gly-Asp	PRP	platelet rich plasma
PPP	platelet poor plasma	ADP	adenosine diphosphate
DMSO	dimethyl sulfoxide	MTT	methyl thiazolyl tetrazolium
PMA	phorbol ester	PGE_1_	prostaglandin E_1_
OD	optical density	FITC	fluorescein isothiocyanate
uPA	urokinase-type plasminogen activator	uPAR	Receptor of the urokinase-type plasminogen activator
BMMY	Basic medium with 2% peptone, 1% yeast extracts, 1.34% Yeast Nitrogen Base without Amino Acids, 1% methanol, 100 mM Potassium phosphate and 4 × 10^−5^ biotin	BMGY	Basic medium with 2% peptone, 1% yeast extracts, 1.34% Yeast Nitrogen Base without Amino Acids, 1% Glycerol, 100 mM Potassium phosphate and 4 × 10^−5^ biotin
RPMI	Roswell Park Memorial Institute		
